# Supplementary figures and images for: Calculated hydration free energies become less accurate with increases in molecular weight
Source: PLoS One. 2024 Sep 19;19(9):e0309996. doi: 10.1371/journal.pone.0309996 (PMC11412681; doi:10.1371/journal.pone.0309996)

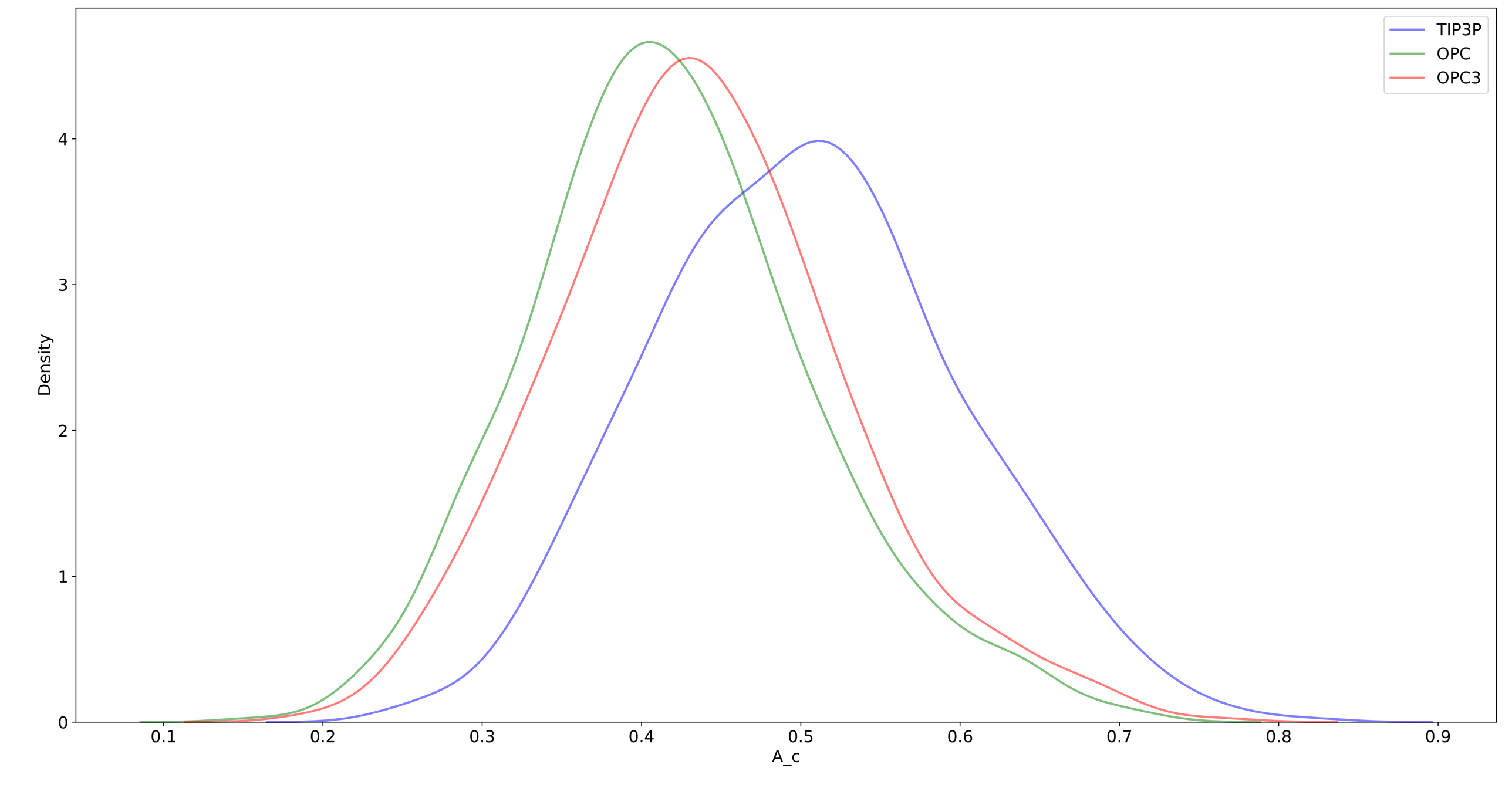

Supplement: S4 Fig — (TIFF) [file pone.0309996.s004.tiff]
