# Supplementary material for: Calculated hydration free energies become less accurate with increases in molecular weight
Source: PLoS One. 2024 Sep 19;19(9):e0309996. doi: 10.1371/journal.pone.0309996 (PMC11412681; doi:10.1371/journal.pone.0309996)
Supplement: Attachment — Submitted filename: 2024-june-hfe-plosOne.pdf [file pone.0309996.s007.pdf]

Review for :
"Calculated hydration free energies become less accurate with increases in molecular weight"

Summary: This article seeks to address and benchmark long-standing problem of how accurately computation is able to predict the hydration free energy (HFE) of drug-like molecules. By using an established dataset of experimentally measured HFE values, the FreeSolv database, the author uses AMBER MD engine to carry out free energy perturbation methods to compute HFE estimations. The author then generates an machine-learned (ML) model trained on this data and assess the performance of said model relative to HFE predictions. The analysis of accuracy focuses on some select molecular properties, such as molecular weight, but does not do a complete benchmarking of other potentially relevant properties. As such, the article could benefit from a greater in-depth discussion on the results, why certain features were chosen, and more discussion on HFE's relevance to drug discovery. As discussed below, other parts require further contextualization, and the discussion section requires a major rewrite as it is a single large paragraph.

Major (required) revisions:

1. The introduction would benefit from a greater direct contextualization of HFE calculations and their relevance in drug discovery. While Free Energy Calculations (FECs) are known to be incredibly relevant to drug discovery and inhibitor improvement, the introduction does not make it clear that HFEs also have their own utility. It would be beneficial if the introduction provided additional citations and additional direct contextualization about where HFEs are valuable in the drug discovery process and what point they're relevant. Obviously, solubility is an important component of the ligand design process, but there is no discussion about additional methods for measuring solubility like logS or how they are utilized.
2. The discussion requires a major rewrite. It is currently one singular large paragraph that spans multiple pages, making it incredibly difficult to read and parse. It is also not clear how the discussion points are connected to the data or are further speculation without the data. It will be important to separate the discussion to clearly demarcate both of those types of paragraphs.
3. Additional discussion on why comparing error to molecular weight and no other properties would benefit the manuscript. In general, I think it is known that HFEs will scale with respect to molecular weight, and with increased ligand size there will be increased parameters to consider and increased time to convergence with simulation-based methods. Thus, it seems consistent with expectation that HFE variance will increase per ligand which would be remedied by increased replicates.
4. Consistent with the above discussion that convergence would require additional sampling with larger molecular weight due to a variety of chemical factors, it would be useful to compare how different numbers of replicates impact this error while still achieving convergence. It would be incredibly useful to identify if there is a scaling for molecular weight, number of atoms/bonds, and the amount of sampling needed.
5. Lastly, it would be useful to provide additional contextualization, testing, and out-of-data comparisons for the Machine Learning model that was constructed. The model tested using these train-test splits never tested against another orthogonal dataset that might be out of distribution. Testing only on data within the dataset allows the model to learn more similar chemistries between the across the train-test split without any guarantee that the model would indeed be able to extrapolate to new chemical topologies.
6. Additionally, a more thorough of the train-test characterization would be useful. It is currently not clear whether the train-test splits contain similar chemical identities in both the training and test sides of the split, which would make it difficult to test how well the dataset is able to extrapolate to new chemistries. Given the importance of the FreeSolv dataset to the ML model, but its small size, it would be useful to do more thorough chemically driven splitting.
7. Given that this was an effort done using established open-source libraries on openly available databases, it would good for there to be an associated github for sharing the results of this data and their weights.
8. Given the interesting nature of the data and the results, a conclusion section would benefit the manuscript and greatly improve readability.

Minor revisions:

1. Citations are needed at the following points:

    a. Page 2, line 31, “an HIV integrase inhibitor”
    b. Page 2, line 32-33, “modeling and simulation have been instrumental in bringing aobut new therapeutic agents”
    c. Page 4, line 86 and 87, “one-step approach” and “two-step approach”
    d. Page 5, line 97, “despite its many known shortcomings”
    e. Page 5, line 96, “if not the most widely used”
2. The following phrases are not clear and could benefit from rewording:
    a. Page 3, line 43-44
3. Start a new paragraph at Page 5 line 108 for clarity.
4. The end of the introduction is riddled with sharp transitions between sentences – some transition words and rephrasing can improve the flow for the reader here.
5. Given the historically outdated nature of the Berendsen Barostat (see papers such as: https://doi.org/10.1016/j.molliq.2022.120116 and https://doi.org/10.1016/j.bbamem.2016.02.004), it would be useful to provide some context for why the Berendsen was used in this simulation over other barostat methods. Alternatively a characterization across different barostats would be useful to see.
6. It would be useful to provide increased contextualization in the text for building the 2D QSAR models
7. Page 8, line 166-167: It would be useful to provide a description what these parameters of zero-variance were that were removed from consideration, and what the other 284 descriptors were in the SI.
8. Page 8, line 169: Please clarify why a 70:30 ratio was chosen for train-test splitting (or provide a citation)
9. Figure would benefit from larger fonts and heading text to improve readability
10. Page 11, line 232: Perhaps a more quantitative description of what it means that the three modes are lying close to each other?
11. Page 14, line 301: Provide a rationale/citation for why 5.25 ns of production dynamics was used, or a more robust sampling was done.
12. Page 15, line 314 appears to have a typo – it should be referring to S5 Fig. if I’m reading correctly?
