# Supplementary material for: Calculated hydration free energies become less accurate with increases in molecular weight
Source: PLoS One. 2024 Sep 19;19(9):e0309996. doi: 10.1371/journal.pone.0309996 (PMC11412681; doi:10.1371/journal.pone.0309996)
Supplement: Attachment — Submitted filename: Response to Reviewers.doc [file pone.0309996.s008.doc]

I thank the reviewers for their positive and constructive comments. Below, I address the concerns they have raised.

Reviewer #1: This manuscript focuses on the impact of molecular properties & choice of water model on hydration free energy – a key component in determination ligand binding free energy. The findings and discussion presented in this study add value to the area of computational drug discovery efforts. I recommend this manuscript for publication after the following comments have been addressed by the author.
1. The author has measured molecular descriptors using RDKit. However, the discussion on the measured descriptors is missing in the discussion. I would recommend adding the list of the descriptors. Did the author find any correlation between one or more of those descriptors and the RMSE in HFE? Did any of those descriptors show correlation with the errors in the sign of the HFE?

I have made the complete set of descriptors and calculated HFEs fully and freely available with the supplementary information to this paper. As suggested by the reviewer, I have explored individual descriptors in the Results and Discussion sections.

2. The results presented in this manuscript show that the 3-point water models outperform the 4-point water model. Could this finding be extended to charged molecules? Should the choice of water model be determined by the physicochemical property of the molecules? A comment on this aspect could be useful if the author has any insights obtained from their analysis.

Currently, alchemical HFE calculations such as the ones we describe here are not capable of handling charged ligands because during decoupling the system’s net charge would change. This introduces large errors of a complex nature; the relevant theory is described in more detail in <https://doi.org/10.1063/1.4826261>. This is why FreeSolv is composed mostly of molecules that have a charge of 0 near neutral pH.

3. While the author has presented an interesting observation regarding the impact of molecular weight on the accuracy of hydration free energy prediction, it will be useful to add a section on why the errors get larger with increase in molecular weight. Specifically, does it have any correlation with the accessible surface area and total volume of the molecule? Do linear molecules show a different trend than cyclic or branched molecules?

I thank the reviewer for this comment, as I feel it is particularly productive. It also aligns with some of the comments made by the other reviewers. Molecular surface descriptors have been explored in more depth in the paper and they do indeed show correlation to the errors in the HFE calculations. Interestingly, the more sophisticated RDKit surface descriptors such as PEOE_VSA and SlogP_VSA have higher correlations than simple descriptors such as total polar surface or total surface area.

Reviewer #2: See Attached Document for formatted review. Plaintext version pasted below:

Review for :
“Calculated hydration free energies become less accurate with increases in molecular weight”

Summary: This article seeks to address and benchmark long-standing problem of how accurately computation is able to predict the hydration free energy (HFE) of drug-like molecules. By using an established dataset of experimentally measured HFE values, the FreeSolv database, the author uses AMBER MD engine to carry out free energy perturbation methods to compute HFE estimations. The author then generates an machine-learned (ML) model trained on this data and assess the performance of said model relative to HFE predictions. The analysis of accuracy focuses on some select molecular properties, such as molecular weight, but does not do a complete benchmarking of other potentially relevant properties. As such, the article could benefit from a greater in-depth discussion on the results, why certain features were chosen, and more discussion on HFE’s relevance to drug discovery. As discussed below, other parts require further contextualization, and the discussion section requires a major rewrite as it is a single large paragraph.

Major (required) revisions:
1. The introduction would benefit from a greater direct contextualization of HFE calculations and their relevance in drug discovery. While Free Energy Calculations (FECs) are known to be incredibly relevant to drug discovery and inhibitor improvement, the introduction does not make it clear that HFEs also have their own utility. It would be beneficial if the introduction provided additional citations and additional direct contextualization about where HFEs are valuable in the drug discovery process and what point they’re relevant. Obviously, solubility is an important component of the ligand design process, but there is no discussion about additional methods for measuring solubility like logS or how they are utilized.

As suggested by the reviewer, I have added an entire paragraph to the Introduction contextualizing HFEs and their relevance in drug design.

2. The discussion requires a major rewrite. It is currently one singular large paragraph that spans multiple pages, making it incredibly difficult to read and parse. It is also not clear how the discussion points are connected to the data or are further speculation without the data. It will be important to separate the discussion to clearly demarcate both of those types of paragraphs.

As suggested by the reviewer, the Discussion has undergone a major rewrite making it shorter and more focused on the work presented here. This comment also nicely aligns with comments by the other reviewers; I have taken this opportunity to add discussion on some of the descriptors to the Discussion section.

3. Additional discussion on why comparing error to molecular weight and no other properties would benefit the manuscript. In general, I think it is known that HFEs will scale with respect to molecular weight, and with increased ligand size there will be increased parameters to consider and increased time to convergence with simulation-based methods. Thus, it seems consistent with expectation that HFE variance will increase per ligand which would be remedied by increased replicates.

I thank the reviewer for this comment as it is particularly constructive. Indeed, my intention for this manuscript was to notify the community of the issue I report and to inspire more investigations into the connection between HFE results (and computational chemistry results in general) and molecular properties. I am well aware that molecular weight is far from the whole story. It is thanks to this comment that I realized that that is never explicitly stated in the text; it is not even suggested. Therefore, I have explicitly stated in the manuscript that it only aims to inspire further analysis; the paper does not claim to be comprehensive. Indeed, this subject can likely fill many more papers-worth of material, likely many books-worth. Here, I have included analysis and discussion on some of the more salient features – weight, number of rotatable bonds, and a few surface area descriptors.

4. Consistent with the above discussion that convergence would require additional sampling with larger molecular weight due to a variety of chemical factors, it would be useful to compare how different numbers of replicates impact this error while still achieving convergence. It would be incredibly useful to identify if there is a scaling for molecular weight, number of atoms/bonds, and the amount of sampling needed.

The reviewer is quite correct to point out that it would be very interesting to see how convergence scales with different factors. Sadly, Matos et al. have not included an estimate for the convergence metric from their 100 ns simulations in the supplementary information of their paper. Therefore, we cannot estimate how convergence scales with simulation time. As for scaling with molecular weight, supplementary figure 5 contains such scatter plots where the R2 between weight and the scaling parameter is around 0.30. However, at this point I am reluctant to make any major claims other than to say there certainly is a relationship. Quantifying it more precisely is the subject of future work, both by myself and other authors. That and the other questions the reviewer poses here would have to be the subject of future investigations as more compute time is presently not available.

5. Lastly, it would be useful to provide additional contextualization, testing, and out-of-data comparisons for the Machine Learning model that was constructed. The model tested using these train-test splits never tested against another orthogonal dataset that might be out of distribution. Testing only on data within the dataset allows the model to learn more similar chemistries between the across the train-test split without any guarantee that the model would indeed be able to extrapolate to new chemical topologies.

I address this comment together with the following one, see below.

6. Additionally, a more thorough of the train-test characterization would be useful. It is currently not clear whether the train-test splits contain similar chemical identities in both the training and test sides of the split, which would make it difficult to test how well the dataset is able to extrapolate to new chemistries. Given the importance of the FreeSolv dataset to the ML model, but its small size, it would be useful to do more thorough chemically driven splitting.

The machine learning section is used only as a story-telling device to introduce the chemical descriptors that will be used to analyze the outcomes from the HFE calculations. Therefore, ML is not the focus of the work.

7. Given that this was an effort done using established open-source libraries on openly available databases, it would good for there to be an associated github for sharing the results of this data and their weights.

All descriptors and HFE results are now included in the supplementary information of this paper for the computational chemistry community to use and analyze. Again, I hope to inspire more papers of this sort, potentially deriving better descriptors, models, and hopefully force fields.

8. Given the interesting nature of the data and the results, a conclusion section would benefit the manuscript and greatly improve readability.

As suggested by the reviewer, a Conclusions section has been added where I explicitly state the goals of this work and its implications.

Minor revisions:
1. Citations are needed at the following points:
a. Page 2, line 31, “an HIV integrase inhibitor”
b. Page 2, line 32-33, “modeling and simulation have been instrumental in bringing aobut new therapeutic agents”
c. Page 4, line 86 and 87, “one-step approach” and “two-step approach”
d. Page 5, line 97, “despite its many known shortcomings”
e. Page 5, line 96, “if not the most widely used”

As requested by the reviewer, all of the above references have been added.

2. The following phrases are not clear and could benefit from rewording:
a. Page 3, line 43-44

This has been simplified.

3. Start a new paragraph at Page 5 line 108 for clarity.

As requested by the reviewer, this is now in a separate paragraph.

4. The end of the introduction is riddled with sharp transitions between sentences – some transition words and rephrasing can improve the flow for the reader here.

The introduction has been rephrased slightly to improve readability.

5. Given the historically outdated nature of the Berendsen Barostat (see papers such as: https://doi.org/10.1016/j.molliq.2022.120116 and https://doi.org/10.1016/j.bbamem.2016.02.004), it would be useful to provide some context for why the Berendsen was used in this simulation over other barostat methods. Alternatively a characterization across different barostats would be useful to see.

Here, I use the Berendsen barostat simply because it is a popular choice and has been a part of our pipeline for a long time. I cannot make any claims about what the results would look like with other barostats, other than to say that I do not expect them to change much. Force fields should be the major determinant of the quality of the calculated results, not the barostat. After all, force fields are the field that is undergoing extensive development, barostats and thermostats – not so much. Presumably, barostats and thermostats are mature enough.

6. It would be useful to provide increased contextualization in the text for building the 2D QSAR models

Again, I do not want to place too much emphasis on the ML section; my primary focus is on the analysis of the HFE results.

7. Page 8, line 166-167: It would be useful to provide a description what these parameters of zero-variance were that were removed from consideration, and what the other 284 descriptors were in the SI.

All descriptors are now available in the SI of the paper.

8. Page 8, line 169: Please clarify why a 70:30 ratio was chosen for train-test splitting (or provide a citation)

70:30 is just a personal preference. Most often researches use 75:25. Indeed, this is the default in many packages, including scikit-learn, and often people use it without even realizing it. I tend to dislike it somewhat because I feel it artificially inflates results – if there is too much data in the training set and too little data in the test set, results may come out looking good simply because there is too little test data to make an error on. However, there is no split that is set in stone, it is entirely up to the user to choose the ratio.

9. Figure would benefit from larger fonts and heading text to improve readability
10. Page 11, line 232: Perhaps a more quantitative description of what it means that the three modes are lying close to each other?

While it would be straightforward to add exact numbers, I believe the text is already fairly heavy on numbers and data. Rather, the goal here is to guide the reader and help them interpret the data rather than to give exact details about the distributions.

11. Page 14, line 301: Provide a rationale/citation for why 5.25 ns of production dynamics was used, or a more robust sampling was done.

The rationale here is quite simple – this is the most I could afford. What is notable is that results differ marginally from 100 ns of sampling, as in the Matos paper (see Fig 5). I stress that the point of the paper is NOT that I can reproduce their results but that my results and their results clearly show a sharp deterioration of performance for larger, more complex ligands. This is something the community will need to acknowledge and address going forward.

12. Page 15, line 314 appears to have a typo – it should be referring to S5 Fig. if I’m reading correctly?

Indeed, the reviewer is correct, this should be S5 Fig. I thank the reviewer for pointing this out.

Reviewer #3: This work compares the experimental Hydration Free Energy (HFE) in the Free Solve dataset to a) values calculated using alchemical free energy methods with thermodynamic integration (TI) estimator and b) to an ML model trained on what I assume is the experimental data. The main conclusion is that the error in the prediction from the alchemical estimation increases with increasing molecular weight.

Overall
There has been considerable effort in producing a useful set of calculations on an important area of computational chemistry, I thank the authors for their efforts. The main points of criticism are that:
1. The implications and reasons for the lack of convergence of the HFE estimates are not adequately explored. This affects the validity of the conclusions drawn.

As requested by reviewers 2 and 3, more discussion on convergence has been added.

2. The inclusion of the ML analysis isn’t fully justified.

This comment aligns with one of the comments from reviewer #2. As I have noted in my response to reviewer #2, the ML section is used only as a story-telling device to introduce the chemical descriptors that will be used to analyze the outcomes from the HFE calculations. Again, ML is not the focus of the work.

3. The discussion areas should be a lot more focused on the topic of the paper.

This comment also nicely aligns with comments from the other reviewers. Indeed, the discussion has undergone a major shortening and rewrite and is now a lot more focused.

4. There should be more references to relevant work.

Relevant references have been added.

There is much here that is interesting and a refocusing of the analysis would be welcome to explore the convergence properties of these calculations.

Introduction
- The introduction is well written and gives an overview of computer aided drug discovery.
- I believe it is too long and not focused enough on the specific area covered by the work and fails to make the case for why this work is necessary.
- The have been numerous works looking at computational predictions of HFE e.g. (non-exhaustive list), The SAMPL challenges or https://doi.org/10.1021/acs.jcim.0c00600, https://pubs.acs.org/doi/abs/10.1021/acs.jcim.0c00285 which should be mentioned.

A paragraph on HFE has been added to the Introduction with proper references, including to SAMPL.

- There have also been many papers on ML methods for QSAR, see https://paperswithcode.com/sota/molecular-property-prediction-on-freesolv for a ‘leaderboard’ of methods on the Free Solv database.
- Other forcefields e.g., CHARMM small molecule forcefield and the recent Open Force Field, Sage 2.0, and Machine Learned forcefields were also not mentioned.

These are now referenced in the text.

Methods
- The methods were mostly clearly explained with the following exceptions: The use of the TI estimator was not fully justified given the success of other estimators and potential drawbacks of TI, e.g., MBAR. See https://pubs.acs.org/doi/abs/10.1021/acs.jcim.0c00285, https://doi.org/10.1063/1.5041835, and https://doi.org/10.1021/acs.jced.7b00104.

TI was used here because it has been a part of our pipeline for some time now. In practice, one should not expect a significant difference between the results from TI or MBAR (or any other variation of the BAR method such as UBAR or RBAR). Indeed, in one of the references the reviewer suggests (<https://pubs.acs.org/doi/abs/10.1021/acs.jcim.0c00285>), the authors conclude that there is no significant difference in performance between TI and MBAR.

- The QSAR variables would be better listed in the SI, rather than given as a reference. While implied, the training data was not explicitly identified as the experimental values. Given there is value and precedence in fitting ML models to ABFE data this should be explicitly mentioned.

All descriptor and HFE data is included in the SI as S1 File. The experimental hydration free energies are now explicitly specified as the target data during training.

- The normalization by the molecular weight, while not wrong, was not justified as inclusion of the molecular weight itself should account for the effect of molecular weight on the predictions and regression coefficients.

I thank the reviewer for this comment. I have expanded upon it in the text. The reasoning behind the normalization is to conduct feature engineering that would hopefully produce descriptors more powerful than the original ones. Also, I hope to inspire future feature engineering efforts by other authors. To that end, I suggest a couple of schemes I believe are worth investigating in the future.

- The fitting procedure was rigorous but the hyperparameter turning curve should be given in the SI.

Given that there are 10,000 models, adding 10,000 curves to the SI would likely be impractical, especially given that the paper is already rather heavy on data and numbers.

- The metric denoted as ‘relative error’ is not consistent with general use of that term (which would include subtraction by 1) please either subtract 1 from the values or use a different term.

I thank the reviewer for their careful examination of my work, but in this case I am unsure what the reviewer is exactly referring to. In any case, the terms here are clearly defined, so any confusion is unlikely. Moreover, the current definition also has the added benefit of being very intuitive – perfectly predicted ligands have relative values of 100%, as opposed to 0%. I believe setting them to 0% would be a lot more confusing to the reader. Also, as things stand now, the x = 0 % axis divides ligands with correctly predicted HFE signs from incorrectly predicted ones. I believe subtracting 100 and shifting that to x = -100% would be less intuitive and more confusing to the reader. Therefore, I believe the current presentation of the data is appropriate.

Results
The results are generally well reported and clear.
- The vertical lines in figure 1 where not explained in the caption.

An explanation has now been added to the captions.

- The discussion of the distribution of prediction errors would benefit from being quantitative (using terms like, bias, standard deviation, kurtosis, etc.) rather qualitative (e.g. ‘TIP3P has the tallest and narrowest error distribution’).

This comment largely coincides with a comment from reviewer #2. While it would be straightforward to add exact numbers, I believe the text is already fairly heavy on numbers and data. Rather, the goal here is to guide the reader and help them interpret the data rather than to specify exact numbers for the distributions.

- In line 236 please convert these values into percentages.

As requested by the reviewer, percentages have been provided, along with the original absolute numbers to remind the reader where these numbers come from and guide them through the text.

- Please clarify what you mean by (line 242) ‘not only do the errors become larger with increasing MW but…’ as the error distribution looks to have an approximate mean of 0 for larger weight. Your note of increasing range and kurtosis looks accurate though.

I thank the reviewer for pointing out this could be a little hard to interpret. Correspondingly, I have expanded this section in the text to make it clear what message I am trying to convey.

- It is hard to draw meaningful conclusions from figure 2 due to its format (scatter plot with different colours). Plotting an estimate of the mean and range / standard deviation etc. of the errors vs MW would be more informative. One could use a Gaussian process, LOWESS smoother or even categorise the molecular weight into ranges and plot box plots. LOWESS smoothers are available in the Seaborn in the regplot function.

The reason for choosing such a visually difficult way of presenting the results is that it allows identifying individual compounds and comparing results across force fields for every single compound. I believe that in the grand scheme of things it is worth the mental effort on the part of the reader to get accustomed to the figure and then have access to all the details in the data, many of which would be lost if the data were to be binned.

- Figure 3 is quite confusing. It would be ideal if you could keep the format of the comparison the same as Figure 2.

There are many more data points from the ML predictions (1,930,000) than the TI predictions (642), so the TI results would hardly be visible in such a plot. Therefore, a density plot is in order. Again, I believe the mental effort on the part of the reader is worth the reward.


Discussion
- I do not believe that the conclusions you draw here are adequately supported by the data. This is because the convergence of the free energy estimates using the FE method drops significantly with increasing molecular weight.
- It’s not clear why MW has been singled out as the factor influencing accuracy given that number of rotatable bonds must also be very influential.

I thank the reviewer for this comment. The present submission aims to alert the computational chemistry community of the following problem: HFE calculations become increasingly inaccurate with increasing molecular size and complexity. Molecular weight is simply one natural descriptor for the size of a molecule. While the paper aims to inspire more work exploring the link between molecular properties and HFE accuracy, I now realize this was never explicitly stated and that the text gives off the impression that I am solely focused on molecular weight. This is not the case. Consequently, I have added results and discussion on other properties as well – number of rotatable bonds and surface area descriptors; I also suggest feature engineering schemes that I believe could provide interesting results in the future.

- I would like to see an analysis of convergence wrt to MW stratified by number of rotatable bonds.

As requested by the reviewer, a breakdown by number of rotatable bonds has been added; I feel this has been particularly useful. The ligands have been grouped in 5 bins – 0 or 1, 2 or 3, 4 or 5, 6 or 7; 8 or more rotatable bonds. A finer breakdown would result in too many bins; I feel 5 panels give sufficient detail without cluttering the figure too much.

- I would also like some investigation into the reasons for the lack of convergence. It’s not clear what you mean by ‘independent’ replicas in line 324. If they are not different configurations, perhaps perform replicas on some of the least converged molecules with different starting configurations.

In this case, “independent” means that minimization, heating, preequilibration, and production dynamics have all been run on the structures provided by FreeSolv in 4 separate replicas, i.e. 4 times. This is important because production runs should start from structures that are as decorrelated as possible. Some authors make the mistake of starting different replicas from the same equilibrated structure and just using random starting velocities. I have made the reader aware that this is not the case here.

I agree that more simulation and convergence analysis would be of much use. However, running more simulations is not an option simply due to budgetary reasons. Therefore, I am forced to draw conclusions from careful analysis of the data I do have – from this work and from other published literature. While Matos et al. have published results based on much longer simulations – around 100 ns per ligand – their results are nearly identical to my own. Again, the point of the paper is NOT that I can reproduce their results with a lot less compute time, it is that my results and their results clearly show a sharp deterioration of calculated HFE accuracies with ligand size. This is one of the main points of the paper, and I believe there is now sufficient evidence, presented in this work and the relevant literature, to make this claim.

- Comparisons between forcefields and water models is valid but only with converged estimates.
- Line 329: sampling multiple short trajectories are only useful if the starting configurations are drawn from the equilibrium configurational distribution (see comment earlier as well).

The reviewer is quite correct; I fully agree with the above.

- In line 362 you say it is not the purpose of the paper to draw definitive conclusions yet the title of the paper is very definitive.

I thank the reviewer for pointing this out. This particular phrase refers to the comparison between GAFF2.11 and GAFF1.81. The manuscript has undergone a significant rewrite and I have tried to soften the tone throughout the text.

- The discussion is very wide ranging and could do with being shortened and restricted to the main points of the paper.

As suggested by the reviewer and reviewer #2, the discussion has been shortened and is now a lot more focused.

- The inclusion of the SVM models in the study was not justified.

The ML is used here as a story-telling device to introduce the molecular descriptors that are used to analyze the results of the HFE calculations – molecular weight, number of rotatable bonds, and surface area descriptors. This is the true focus of the paper, not the ML.
